# Association of Exposure to Communities With High Obesity With Body Type Norms and Obesity Risk Among Teenagers

**DOI:** 10.1001/jamanetworkopen.2020.0846

**Published:** 2020-03-16

**Authors:** Ashlesha Datar, Amy Mahler, Nancy Nicosia

**Affiliations:** 1 Center for Economic and Social Research, University of Southern California, Los Angeles; 2 Department of Economics, University of Southern California, Los Angeles; 3 RAND Corporation, Boston, Massachusetts

## Abstract

This cross-sectional study examines whether teenagers’ exposure to communities with high obesity rates is associated with ideal body type and risk of obesity.

## Introduction

There is substantial evidence of clustering of obesity within geographic and social networks.^1^ Designing effective policies requires understanding whether this clustering is owing to shared environments, homophily, or social contagion. However, conclusive evidence is limited by the lack of quasi-experimental or natural experiment studies.

In a 2018 study,^2^ we analyzed data from a natural experiment, the Military Teenagers Environments, Exercise, and Nutrition Study (M-TEENS), in which US Army families were exposed to communities with varying rates of obesity as a result of their assignments to specific installations. We found that teenagers assigned to counties with higher obesity rates were more likely to have overweight or obesity. The study design ruled out homophily as an explanation, and we found no evidence that shared environments explained these results. We suggested that our findings may be consistent with social contagion with the goal of exploring this issue in future work. In the current cross-sectional study, we used newly collected M-TEENS data to examine whether teenagers’ exposure to communities with high obesity was associated with their ideal body type and obesity risk.

## Methods

The study followed the Strengthening the Reporting of Observational Studies in Epidemiology (STROBE) reporting guideline for cross-sectional studies. It was approved by the University of Southern California institutional review board.

Between Dec 2017 and July 2018, 401 M-TEENS participants (aged 16-19 years) and their parents completed online surveys that included self-reports and parent-reports regarding teenagers’ height, weight, and other individual and household covariates. Parent consent and child assent were obtained online. Height and weight measurements, available for approximately half the sample, were used to correct bias in self-reports and parent-reports using regression calibration. Adolescents were classified as having obesity if this corrected body mass index, calculated as weight in kilograms divided by height in meters squared, was in at least the 95th percentile for their age and sex.

Adolescents selected their ideal body type (IBT) on a figure rating scale consisting of 9 figures, matched by their sex, that captured normal body mass index (figures A-C), overweight (figure D), and obesity (figures E-I).^3^ We focused on whether they chose an IBT that reflected overweight or obesity, defined as figures D through I.

County obesity rates (COR) for their current installations were obtained from the Robert Wood Johnson Foundation County Health Rankings Data.^4^ In line with prior research suggesting a tipping point in social norms,^5^ we captured high obesity exposure via an indicator for whether the COR exceeded the median (ie, 30.5%). We hypothesized that teenagers exposed to counties with high obesity rates would tend to choose an IBT reflecting overweight or obesity because of changes in their descriptive and injunctive norms about body size and would have higher obesity risk.

Multivariable logistic models estimated the association between exposure to high COR and selecting an IBT reflecting overweight or obesity and then between high COR and obesity, adjusting for IBT. All analyses were conducted in Stata version 14 (StataCorp). No prespecified level of statistical significance was set.

## Results

Sample characteristics are reported in Table 1. Teenagers exposed to high COR had significantly higher odds of choosing an IBT reflecting overweight or obesity compared with those exposed to low COR (adjusted odds ratio, 1.96; 95% CI, 1.18-3.24) (Table 2). The odds of having obesity among teenagers exposed to high vs low COR were 2.33 (95% CI, 1.09-4.98) without adjusting for IBT and decreased to 1.75 (95% CI, 0.79-3.88) after adjusting for IBT. However, the decrease was not statistically significant.

**Table 1.** Characteristics of 401 Participants

Measure	No. (%)
Obesity status	
Obesity	57 (14.2)
No obesity	344 (85.8)
Exposure	
Installation county obesity rate, mean (SD) [range], %^a^	30.0 (4.1) [18.3-37.1]
High county obesity rate	226 (56.4)
Ideal body type figure^b^	
A	6 (1.5)
B	75 (18.7)
C	204 (50.9)
D	106 (26.4)
E	7 (1.8)
F	3 (0.8)
>C	116 (28.9)
Covariates	
Participant characteristics	
Age, mean (SD), y	17 (0.67)
Men	220 (54.9)
Women	181 (45.1)
Race/ethnicity	
Non-Hispanic white	158 (39.4)
Non-Hispanic black	84 (20.9)
Hispanic	99 (24.7)
Other race/ethnicity^c^	60 (15.0)
Parent characteristics	
Military service member	232 (57.9)
Active duty family	238 (59.4)
Parent married	355 (88.5)
Military rank ≥E7, ie, sergeant first class	189 (47.1)
Highest education level of parents	
≤Trade or technical school	30 (7.5)
Some college	184 (45.9)
≥4-y college degree	187 (46.6)
Family characteristics	
Annual household income ≥$70 000	141 (35.2)
Time at installation ≤24 mo	141 (35.2)
No. of children in household, mean (SD)	2.4 (1.2)
Family lives off installation	300 (74.8)
County built environment^d^	
Food environment index, mean (SD)^e^	6.7 (1.2)
Population with access to exercise opportunities, mean (SD), %	78.1 (13.0)

^a^ Our sample was spread across 59 installations in 57 counties.

^b^ Figures selected from a figure rating scale in which figures A through C captured normal weight, figure D captured overweight, and figures E through I captured obesity. No participants chose ideal body type figures G to I.

^c^ Other category includes Asian, American Indian or Pacific Islander, and individuals who selected multiple races/ethnicities.

^d^ County built environment measures came from the Robert Wood Johnson Foundation County Health Rankings data.

^e^ Food environment index accounts for both proximity to healthy foods and income of county residents, ranging from 0 (worst) to 10 (best).

**Table 2.** Association of Exposure to High Obesity County With Norms Regarding Ideal Body Type and Obesity Risk^a^

Explanatory Variable	aOR (95% CI)		
	Model 1, Ideal Body Type	Model 2, Obesity	Model 3, Obesity
County obesity rate			
Low	1 [Reference]	1 [Reference]	1 [Reference]
High^b^	1.96 (1.18-3.24)	2.33 (1.09-4.98)	1.75 (0.79-3.88)
Ideal body type			
A-C	NA^c^	NA^c^	1 [Reference]
>C	NA^c^	NA^c^	9.83 (5.28-18.29)

Abbreviations: aOR, adjusted odds ratio; NA, not applicable.

^a^ Results for 401 participants. All models control for covariates described in Table 1. Model 1 estimated the association of exposure to high county obesity rate with ideal body type reflecting overweight or obesity. Model 2 estimated association of high county obesity rate with having obesity. Model 3 estimated association of high county obesity rate with having obesity, adjusted for ideal body type. Standard errors were adjusted for clustering of sample within installations.

^b^ High county obesity rate indicates that the county obesity rate was greater than the median (ie, >30.5%).

^c^ These cells are blank because the model did not adjust for ideal body type.

## Discussion

Teenagers’ exposure to high obesity was associated with selecting an IBT reflecting overweight or obesity and greater obesity risk. These findings suggest that high obesity rates may normalize unhealthy weight,^6^ making obesity prevention more difficult. While exposure to high obesity rates primarily because of military parents’ installation assignment was a key strength of the study, the cross-sectional design and generalizability were limitations. Longitudinal data on IBT norms and obesity risk from future M-TEENS waves can help assess the temporal association of obesity exposure with IBT norms and risk of obesity among teenagers.
